# Predictive Performance of Bayesian Methods to Forecast Vancomycin Concentration for Therapeutic Drug Monitoring in Critically Ill Pediatric Patients

**DOI:** 10.3390/pharmaceutics18020160

**Published:** 2026-01-26

**Authors:** Ha T. Pham, Cuc T. Nguyen, Tien T. N. Nguyen, Linh H. Hoang, Minh N. Tran, Thao P. Nguyen, Tuan N. Do, Ha T. H. Nguyen, Anh H. Nguyen, Phuc H. Phan, Dien M. Tran, Hoa D. Vu

**Affiliations:** 1 Vietnam National Children’s Hospital, Hanoi 11500, Vietnam; hapt@nch.gov.vn (H.T.P.); nhatminh@nch.gov.vn (M.N.T.); dientm@nch.gov.vn (D.M.T.);; 2 National Drug Information and Adverse Drug Reaction Monitoring Centre, Hanoi University of Pharmacy, Hanoi 11021, Vietnam; cucnt@hup.edu.vn (C.T.N.); linhhh@hup.edu.vn (L.H.H.); ngthao.20107@gmail.com (T.P.N.);; 3 N2TP Technology Solutions JSC, Hanoi 11600, Vietnam

**Keywords:** vancomycin, therapeutic drug monitoring, critically ill, pediatric, Bayesian forecasting

## Abstract

**Background:** This study aimed to evaluate different Bayesian algorithms and the first-order pharmacokinetics (PK) equation approach for forecasting vancomycin concentrations in critically ill pediatric patients and to identify influencing factors. **Methods:** A cohort of 110 patients with 568 therapeutic drug monitoring (TDM) blood samples was included. Three Bayesian algorithms, i.e., conventional, flattened, and weighted-flattened, using one or two historical values of either blood concentrations measured at the peak, trough, or middle (mid) of the dosing interval, were applied to forecast the concentrations of the next TDM occasion. The first-order PK approach, according to the Sawchuk–Zaske method, was used with two levels. The forecasting performance was assessed via relative bias (rBias) and relative root mean squared error (rRMSE) between the forecasted and observed levels. A linearmixed-effects model was employed to identify potential influencing factors on the rBias and rRMSE. **Results:** All methods showed negative rBias values of less than −20% and had relatively similar rRMSE of about 40%. First-order PK had lower bias than the conventional and flattened Bayesian algorithm (−10% vs. −15%), but higher bias than the weighted-flattened Bayesian algorithm (rBias −5%). Multivariate analysis using the linear mixed-effects model revealed that the type of forecasting algorithms significantly impacted the predictability. The weighted-flattened Bayesian algorithm significantly improved the rBias by 12.660% (95% CI: 10.131–15.194, *p*-value < 0.001) and decreased the rRMSE by 2.099% (CI 95% 3.779–0.418, *p*-value = 0.014) compared to the conventional Bayesian model. Either using one (mid or trough) or two concentrations in Bayesian forecasting yielded comparable rBias and rRMSE. **Conclusion:** The weighted-flattened Bayesian estimation method with solely one blood level is appropriate for forecasting the vancomycin concentration during therapeutic drug monitoring in critically ill children.

## 1. Introduction

Vancomycin is an important antibiotic used for suspected or documented infections caused by methicillin-resistant *Staphylococcus aureus* (MRSA), one of the most common multidrug-resistant pathogens in pediatric patients [1]. Pharmacokinetics (PK) profiles of vancomycin showed high variability in this population due to the continuous growth in body size and organs, the complex clinical conditions, and intensive intervention [2,3]. Taking into account the narrow therapeutic range of vancomycin, therapeutic drug monitoring (TDM) has been recommended to ensure efficacy and minimize the risk of toxicity, including acute kidney injury (AKI) [4,5,6].

Trough-guided dosing was initially recommended because of its convenience in clinical settings [1,5]. However, recent evidence showed that trough concentration alone had not been an appropriate surrogate of vancomycin exposure, and trough-based TDM might put patients at higher risk of nephrotoxicity [4,7,8]. Therefore, the updated consensus guidelines on vancomycin TDM have advocated the usage of the area under the curve (AUC) for dosing recommendations, supported by specialized software [4,9,10]. Herein, AUC was estimated via the first-order PK equation with at least two concentrations or Bayesian approaches [4,11,12,13]. The latter, relying on an a priori population pharmacokinetics (popPK) model and observed data, i.e., vancomycin concentration(s) of each individual, recently became a potential solution to tailor dosage regimens [14,15,16]. It applies Bayes’ rules to estimate the individual PK parameters, which were then used to predict the PK profile and forecast unmeasured vancomycin concentrations. However, the utilization of one or two or even more TDM samples for Bayesian forecasting remains controversial. It should be noted that using a minimum number of TDM samples to accurately and precisely forecast vancomycin concentration and respective drug exposure benefits vulnerable populations such as critically ill neonates and pediatric patients. Therefore, several Bayesian algorithms and proposed approaches to simplify the sampling schedule were developed [17,18]. However, these studies were only conducted in hospitalized adult patients. It is questioned whether this novel approach could produce reliable forecasting performance in critically ill pediatrics in comparison to conventional methods.

This study aimed to evaluate and compare the forecasting performance between the first-order PK method and various Bayesian algorithms, using one and two TDM samples of vancomycin in critically ill pediatric patients.

## 2. Methods

### 2.1. Study Design and Setting

This study was retrospectively conducted in the pediatric intensive care unit (ICU) department of multispecialty and tertiary referral children’s hospital in the North of Vietnam (Vietnam National Children’s Hospital). Vancomycin was primarily prescribed in the empirical antibiotic regimen for MRSA. The initial dose of vancomycin was determined according to an established dosing nomogram, depending on age and estimated glomerular filtration rate (eGFR) using the Schwartz equation [19]. The dose was then titrated as needed to achieve the AUC target of 400–600 mg·h/L following approved institutional TDM protocol. The blood samples were collected at least 24 h after the initial dose, and repeated after dose adjustment if the AUC target was not attained. The vancomycin concentration measurements were also conducted in several circumstances, such as with patients with unstable renal function or other sudden changes. Herein, AUC values were calculated via the Sawchuk–Zaske method, using two concentrations: peak concentration (Cpeak) at 1–2 h after the end of the infusion and trough concentration (Ctrough) at 0.5–1 h prior to the next dose [4,13]. The sample not belonging to these time windows was considered mid-concentration (Cmid) and was also used to estimate AUC for TDM. The study was approved by the Human Research Ethics Committee of the study hospital (Approval No. 2508/BVNTW-HDDD). Due to the retrospective nature, informed consents were waived.

### 2.2. Data Collection

The data of pediatric patients with AUC-guided TDM indication of vancomycin from December 2022 to January 2024 were thoroughly screened from medical records. Children aged from 3 months to 18 years who received vancomycin via intermittent intravenous infusion, with two post-dose concentrations and at least one concentration collected at another following dose, were included. Patients were excluded if their medical records were inaccessible or if they underwent any extracorporeal circulation interventions during the vancomycin TDM period (e.g., dialysis, plasma exchange (PEX), or extracorporeal membrane oxygenation (ECMO)).

The data were extracted and filled into the data collection form (Table S1, Supplementary) by one investigator and then inspected independently by others. The extracted information included age (day), gender, total body weight (kg), height (cm), baseline serum creatinine (Scr, mg/dL), eGFR (mL/min/1.73m^2^), mechanical ventilation support, septic shock, infectious disease, concurrent use of agents, vancomycin dosage regimen (mg, dosing interval), sampling time (h), vancomycin concentration (mg/L), length of vancomycin therapy (day), and length of ICU stay (day). Vancomycin quantification was performed by an automation-ready immunoassay and clinical chemistry analyzer (Atellica CH930 module, Atellica^®^ Solution, Siemens Healthcare Diagnostics Inc., New York, NY, USA).

### 2.3. ForecastingMethods

**Figure 1:**
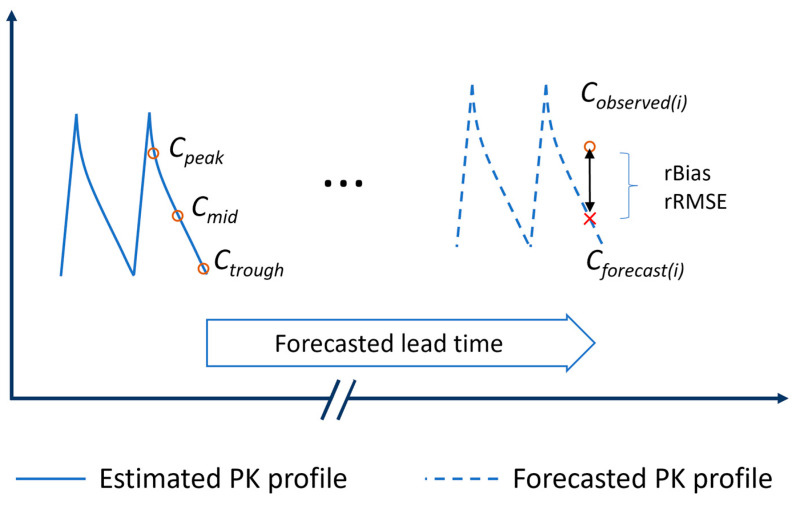
Forecasting methods. // represents any predicted lead time that varies between patients or predictions.

The forecasting performance of various methods, including the first-order PK calculation and Bayesian estimation versions, was examined (Figure 1). In the first-order PK calculation, the Sawchuk–Zaske method with two blood levels was applied to identify the pharmacokinetic profile, assuming steady-state had been attained [13]. Herein, the two post-dose concentrations at the preceding TDM occasion were used to estimate the constant of elimination rate (k_e_), volume of distribution (V_d_), and present the individual concentration-time profile. Based on these PK parameters, the sampling time at the subsequent TDM occasion was integrated to forecast the respective concentrations (Cforecast(i)). In the Bayesian method, a previously published one-compartmental popPK model of vancomycin in children by Le et al. (2014) was used as a priori information [20]. Bayesian estimation was then performed using one or both drug concentrations of the preceding TDM occasion as evidence to estimate individual PK parameters as posterior and simulate the forecast concentration-time profile. The sampling time at the subsequent TDM occasion was added in order to forecast the next vancomycin concentrations. The modifiable variables, including dose and serum creatinine updated in the forecast lead time, were also taken into account. The forecasting performance was evaluated by comparing the forecasted and the respective observed vancomycin concentrations in the subsequent TDM. Two metrics, including relative bias (rBias) and relative root mean squared error (rRMSE), were examined according to Equations (1) and (2) below [21,22]:

(1) $$\mathrm{rBias}\;=\;\frac{1}{n}\sum_{i=1}^{n}\frac{Cforecast(i)-\;Cobserved(i)}{(Cforecast(i)+Cobserved(i))/2\;}\left(\%\right)$$

(2) $$\mathrm{rRMSE}=\sqrt{\frac{1}{n}\sum_{i=1}^{n}{\left(\frac{Cforecast(i)-Cobserved(i)}{(Cforecast(i)+Cobserved(i))/2\;}\right)}^{2}}\left(\%\right)$$

where n represents the number of observations of drug levels at the subsequent TDM occasion, while i refers to the ith observed concentration among these n values. The Cobserved(i) is the ith observed concentration, and Cforecast(i) is the ith corresponding forecasted concentration.

For the Bayesian estimation, we tested three versions. First, the conventional Bayesian approach was performed using standard Maximum A Posteriori (MAP) estimation in NONMEM version 7.5.0 (ICON Development Plc, South Country Business Park, Leopardstown, Dublin 18, Ireland) [16,23]. The following objective function (OBJBayes) (3) was minimized:

(3) $$OBJBayes=\;\sum_{j=1}^{m}\frac{{\left(Cobserved(j)\;-\;f(\theta ,tj\right))}^{2}}{{\sigma j}^{2}}+\sum_{k=1}^{s}\frac{{{(\theta }_{k}\;-\;{\theta^{\prime }}_{k})}^{2}}{\omega_{k}^{2}}$$

m: The number of observations of each individual at the preceding TDM occasion;

j: The jth observed concentration of each individual among these m values;

Cobserved(j): The jth observed concentration;

θ: The vector of the pharmacokinetic parameters of the model (clearance, volume of distribution, etc.);

Tj: The sampling time of the jth observed concentration;

F(θ,tj): The jth model-predicted concentration;

σj: Residual error variance on Cobserved(j);

s: The number of pharmacokinetic parameters;

k: The kth pharmacokinetic parameter;

θk: Population parameter values;

θ′k: Individual’s estimated parameter values;

ωk: Parameter residual variances.

Second, the flattened Bayesian approach was employed following Hughes et al., by using flattened coefficients (flat_coef) as a weight to control the contribution of a priori (i.e., popPK model (Equation (4)). The flat_coef of 0.005, 0.02, 0.125, 0.2, 0.3, and 0.6 were tested [17].

(4) $$OBJBayes=\sum_{j=1}^{m}\frac{{\left(Cobserved(j)-f(\theta ,tj\right))}^{2}}{{\sigma j}^{2}}+flat\_coef*\sum_{k=1}^{s}\frac{{{(\theta }_{k}\;-\;{\theta^{\prime }}_{k})}^{2}}{\omega_{k}^{2}}$$

Finally, a novel weighted flat_coef, as presented in Equation (5), was introduced using our in-house developed model-informed precision dosing software (SmartDose^AI,^, version 0.9.0-beta). The software is currently applied for the TDM of vancomycin in a number of hospitals in Vietnam [24,25,26,27,28]. The selection of an appropriate flat_coef parameter is now a critical step that is undertaken during each instance of Bayesian optimization. This process is defined by the equation

(5) $$weighted\;flat\_coef={\;ML(C}_{observed(j)\;},\;f(\theta ,tj),\;clinical\_info)$$

Herein, weighted_flat_coef was selected from a continuous range between 0 and 1 for each patient. ML was a machine learning model that had been trained on historical data within the SmartDose^AI^ system. The independent variables for this model included observed concentrations, a concentration predicted by the popPK model, and the clinical information (clinical_info) of the patient. The dependent variable was the optimal flat_coef, which was determined to most accurately forecast the next observed concentration. The weighted_flat_coef is not fixed during the treatment of a patient. It varies depending on each decision-making occasion of dose adjustment based on available TDM data. Following the calculation of the weighted_flat_coef, the objective function is modified accordingly:

(6) $$OBJBayes=\;weighted\_flat\_coef*\sum_{j=1}^{m}\frac{{\left(Cobserved(j)\;-\;f(\theta ,tj\right))}^{2}}{{\sigma j}^{2}}+(1\;-weighted\_flat\_coef)*\sum_{k=1}^{s}\frac{{{(\theta }_{k}\;-\;{\theta^{\prime }}_{k})}^{2}}{\omega_{k}^{2}}$$

In each Bayesian forecasting method, a single concentration (Cpeak, Cmid, or Ctrough) or both concentrations (Cpeak and Cmid, Cpeak and Ctrough, or Cmid and Ctrough) from preceding TDM times were examined to forecast the future concentration (Figure 1). The model control streams applied for versions of Bayesian methods were available in the Supplementary Information.

### 2.4. Statistical Analysis

A non-parametric bootstrap with 1000 resample was applied to generate a set of forecasted concentrations, and 95% confidence intervals (95% CI) of rBias and rRMSE were derived. Linear mixed-effects regression analysis with random intercept was also used with rBias and rRMSE as dependent variables to examine the determinants of forecasting performance. The independent variables included the type of forecasting methods (using the conventional Bayesian estimation as the reference), the type of measured concentrations in the forecast (Cpeak, Cmid, or Ctrough versus two-point measurement), the forecast lead time, and other patient characteristics such as age, body weight, and serum creatinine. An univariable model was applied first to identify significant variables. Only significant variables identified in the univariable model were introduced into the multivariable model to build the final model. A *p*-value of 0.05 was considered a significant cut-off unless otherwise stated. Statistical analyses were performed using the R language, version 4.5.0 (R-project.org, R Development Core Team, Vienna, Austria). The *ggplot2* package (version 3.5.2) was used for data visualization purposes, and the linear mixed-effects regression was performed using the *lme4* package (version 1.1-37) in R version 4.5.0.

## 3. Results

### 3.1. Patient Characteristics

A total of 110 patients were eligible for this study. Patient characteristics and TDM-related information are described in Table 1. Our cohort had 58 (53.6%) males, age IQR from 0.6 to 4.7 years, and a median weight of 10 (IQR 7–15) kg. The patients ranging from 3 months to 1 year accounted for 37.3%, and the median age and IQR of this subgroup were 5.4 and 3.7–8.3 months, respectively. The median baseline serum creatinine was 0.34 (IQR 0.22–0.49) mg/dL. Approximately 66.1% and 44.6% of the patients suffered from nosocomial pneumonia and bloodstream infection, respectively.

### 3.2. Vancomycin Dosing and Concentrations

**Figure 2:**
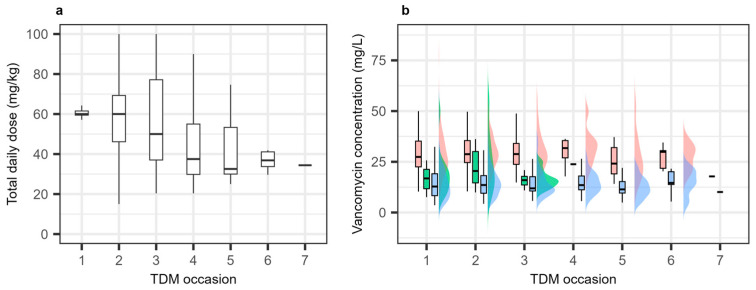
Dosing (**a**) and therapeutic drug monitoring (**b**) data. Red color represents Cpeak, green color represents Cmid, and blue color represents Ctrough.

The median starting total daily dose of vancomycin was 60 mg/kg, with a narrow dose range, i.e., IQR 59.6–61.9 mg/kg. The dose range increased substantially during treatment, from about 20 to 100 mg/kg (Figure 2a). A total of 568 samples were collected (mean 5 samples per patient), with 220 measurements at the first TDM and 348 measurements collected during subsequent TDM occasions. Of these, Cpeak, Cmid, and Ctrough were collected at a median of 2 h (IQR 1.93 h–2.10 h), 4.72 h (IQR 3.59 h–5.40 h), and 5.5 h (IQR 5.48 h–5.82 h), respectively. Significant inter-individual variabilities (IIVs) were observed in peak, mid, and trough levels at all TDM occasions (Figure 2b).

### 3.3. Performance of Different Forecasting Methods

**Figure 3:**
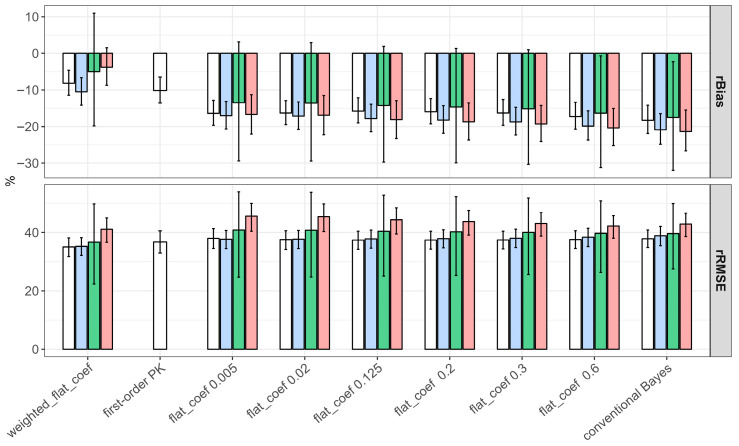
rBias and rRMSE between the forecasted versus the observed vancomycin concentrations based on the weighted-flattened Bayesian, first-order PK, the flattened Bayesian, and the conventional Bayesian approaches. The white color represents two concentrations, the red color represents Cpeak, the green color represents Cmid, and the blue color represents Ctrough.

All forecasting methods had negative rBias values, suggesting that the forecasted concentrations were likely lower than the measured concentrations (Figure 3). The rBias of first-order PK methods (−10%) was smaller than that of conventional and flattened Bayesian methods (about −15%), but relatively larger than that of weighted-flattened Bayesian methods (rBias −5%). We also observed that a smaller flat_coef in a flattened Bayesian resulted in improving the forecasting bias. However, the rRMSE was comparable between methods (about 40%). Either the use of one or two concentrations in Bayesian forecasting produced comparable rBias and rRMSE values, in which peak-based Bayesian forecasting showed a relatively higher rRMSE value. We also observed that Bayesian forecasting using mid-concentration showed the highest variability in predictive performance, i.e., a wide 95% confidence range for rBias and rRMSE (Figure 3).

A statistically significant association between the rBias and the forecasting algorithm was also revealed by linear mixed-effects modeling (Table 2). The first-order PK equation and the weighted-flattened Bayesian approach significantly increased the rBias by 8.932% (95% CI: 5.242–12.623% and *p*-value < 0.001) and 12.660% (95% CI: 10.131–15.194% and *p*-value < 0.001) compared to the conventional Bayesian approach. In addition, the weighted-flattened Bayesian model reduced the rRMSE by 2.099% (95% CI: 3.779–0.418% and *p*-value = 0.014), as presented in Table 3. Using two samples added no further significant benefit in forecasting performance than using one concentration only. In addition, the forecast lead time to the next TDM showed a negative effect of predictability, in which a 1-day increment decreased rBias by 3.311% (95% CI: 2.720–3.902% and *p*-value < 0.001) and increased rRMSE by 3.227% (2.834–3.621%) (Table 3).

## 4. Discussion

This study examined various forecasting methods of the vancomycin level in critically ill pediatric patients, which is a special subpopulation needing to minimize the sampling intervention. Bayesian methods, whether using one or two concentrations, showed non-inferiority performance with the first-order PK method. Furthermore, the weighted-flattened Bayesian estimation demonstrated an advance in the forecast performance, holding potential to integrate into the model-informed precision dosing (MIPD) platform for vancomycin TDM. This approach takes into account the balance between the weight contribution of the prior model and the collected TDM data in the likelihood function to estimate individual PK parameters. The forecast lead time was identified as a key factor influencing the predictive error. To the best of our knowledge, these were not investigated in any published studies.

The Bayesian-based method allows for the integration of time-varying patient characteristics such as age, weight, renal function, and other sudden changes in covariates that affect PK parameters, for dosing recommendations [14,15,16]. Therefore, this method is advantageous for special populations, especially critically ill pediatric patients [14]. In a study using TDM samples from pediatrics, Bayesian estimation using two measurements showed higher accuracy and precision than one sample [20]. In another study, although two samples were necessary for first-order PK calculation, Bayesian estimation using trough measurement was also sufficient and reliable [29]. Notably, these studies reported the Bayesian prediction of AUC24h rather than forecasting the concentration of the upcoming doses. The question about the next TDM concentration forecasting remains limited. The forecasting performance depends not only on the mathematical algorithm but also on the change in patient pharmacokinetic characteristics during treatment.

With the development of pharmacometrics and its application, studies to optimize the Bayesian algorithm and attempt to simplify the sampling schedule were implemented [18]. In addition, an innovative method to enhance the performance of Bayesian forecasting through the “flattened priors” approach was introduced [17]. This algorithm allows for reducing the weight of the prior popPK model, and puts more weight on observed patients’ data in PK parameter estimation [17]. Our study used the prior popPK model reported by Le et al. This model, developed in a large pediatric patient population and with the intensive sampling strategy, is relatively generalized for our intended population [20]. However, it is of note that in clinical practice, patients may belong to multiple subpopulations or have high PK variability. In several circumstances, popPK models, a priori, do not describe patient PK characteristics well due to the limited sampling strategy or outliers. Hughes et al. (2022) developed this algorithm to deal with this scenario on a large scale of adult patients with the flat_coef ranging from 0.02 to 0.6 [17]. Accordingly, with the well-performed popPK models, the flat_coef of 0.3–0.6 produced the lowest prediction error. The higher coefficient of greater than 0.6 may not significantly reduce the predictive error compared to the conventional MAP approach, i.e., the flat_coef of 1. In our pediatric population, this method improved Bayesian forecasting performance significantly by a reduction in rBias compared to the conventional method, and was reaffirmed in the precision as rRMSE. In addition, we observed a reduced trend of rBias when the flat_coef decreases. Therefore, a lower flat_coef of 0.005 was tested, and a negligible reduction in rBias was detected. Further reduction was unnecessary to improve the forecasting accuracy. Importantly, we introduced the weighted-flattened Bayesian method, which showed good performance in improving systemic deviation, as evidenced by the reduction in rBias. The rRMSE values imply that the residual error decreased modestly. This implies that individual PK variability during the treatment was very high. In this study, we observed that the forecast lead time is a significant factor predicting elevated concentration with a regression coefficient of rBias of −3.311 (*p*-value < 0.001) and resulting in less precision with a regression coefficient of rRMSE of 3.227 (*p*-value < 0.001). This emphasized the importance and frequency of monitoring the blood level of vancomycin in long-term treatment to avoid the accumulation of the drug in the blood and therefore reduce the risk of kidney injury [30]. The results showed that the trough or mid sampling scheme was non-inferior to the two-point sampling scheme. However, no similarity could be created when using the peak sampling scheme. The peak-based approach overestimated prospective concentrations. It might stem from the situation that peak concentration is governed by the distribution volume, thereby providing a lack of clearance information for each individual. The mid or trough approach is also modest in information after the end of infusion, yet it generated reliable estimates about the concentration-time profile.

TDM should be performed with minimal invasion on patients and with a simple sampling procedure to improve adherence to the practical protocol [31]. For pediatrics, this is a special request from the clinical setting to avoid burdening such a vulnerable population [32]. In our study, a single blood sample in combination with a novel Bayesian estimation demonstrated non-inferior forecasting performance in comparison with the two-blood-sample protocol. The accuracy and precision of the single-sample forecast were observed with minor differences among sampling times. It suggested that robust results could be obtained with the proposed weighted-flattened Bayesian method regardless of the flexibility in sampling time. It is of note that the convenience of blood sampling is an important factor that could improve and facilitate TDM practice [18,31].

There are some limitations in our study. First, our limited sample size might prevent the robustness of the final conclusion; further studies are warranted for validation. Second, the study population was in an extreme setting. Therefore, findings should not be extrapolated to less seriously ill children, as this population exhibits different PK profiles of vancomycin. However, we considered this population to be the group most in need of optimized treatment. Third, this study did not evaluate albumin due to the insufficient retrospective data. In addition, the potential impact of albumin level on vancomycin PK in pediatric patients is still debated [33,34,35]. Fourth, assessing other popPK models was beyond the aim of this study. We focus on testing different methods to forecast the next concentration in TDM. This study plays the role of an external validation for the popPK model integrated into the MIPD software (SmartDose^AI^, version 0.9.0-beta) and other Bayesian algorithms applied. Finally, we evaluate the forecasting performance based on the concentration, which does not necessarily reflect the total exposure of vancomycin. Nevertheless, MIPD applying the Bayesian method requires the measured concentration as an input for the estimation of vancomycin exposure, and therefore, the forecast levels will play an important role in predicting the efficacy and toxicity of the treatment.

## 5. Conclusions

Modification of the Bayesian algorithm, namely the weighted-flattened Bayesian algorithm, showed an improvement in forecasting performance in therapeutic drug monitoring. In conclusion, our study demonstrated the advantages of the weighted-flattened Bayesian method by using one vancomycin concentration to integrate into the model-informed precision dosing platform for critically ill children.

## Figures and Tables

**Table 1:** Characteristics of patients and vancomycin administration.

Characteristics	Value (N = 110)
***Patient characteristics***	
Age (year), median (IQR)	2.0 (0.6–4.7)
<1, no. (%)	41 (37.3)
1 to <2, no. (%)	14 (12.7)
2 to <12, no. (%)	49 (44.5)
≥9, no. (%)	6 (5.5)
Male gender, no. (%)	58 (53.6)
Total body weight (kg), median (IQR)	10.0 (7.0–15.0)
Baseline serum creatinine (mg/dL), median (IQR)	0.34 (0.22–0.49)
eGFR (mL/min), median (IQR)	113.0 (82.7–178.0)
Mechanical ventilation support, no. (%)	96 (87.3)
Septic shock, no. (%)	44 (41.1)
Infectious disease, no. (%)	
Nosocomial pneumonia	73 (66.1)
Bloodstream infection/bacteremia	49 (44.6)
Meningitis	10 (8.9)
Others	5 (4.5)
Concurrent use of agents, no. (%)	
Furosemide	93 (84.5)
Vasopressor drugs	82 (75.5)
Other nephrotoxic agents	45 (40.9)
***Vancomycin administration***	
Empirical total daily dose (mg/kg), median (IQR)	60.0 (59.6–61.9)
Initial dosing interval, no. (%)	
Every 6 h	91 (82.7)
Every 8 h	17 (15.5)
Other (12 h, 24 h)	2 (1.8)
Total concentrations, no. (%)	568 (100.0)
The first TDM	220 (38.7)
The subsequent TDM occasion	348 (61.3)
Time after dose to vancomycin concentration (h), median (IQR)	
Peak concentration	2.0 (1.93–2.10)
Mid concentration	4.72 (3.59–5.40)
Trough concentration	5.5 (5.48–5.82)
Length of vancomycin therapy (days), median (IQR)	13.0 (8.0–18.8)
Length of ICU stay (days), median (IQR)	13.0 (8.0–24.5)

**Table 2:** Results of the linear mixed-effect regression analysis for the dependent variable rBias.

Variables	Unadjusted			Adjusted		
	Estimate	95% CI	*p*	Estimate	95% CI	*p*
**Age (month)**	−0.100	−0.209 to 0.009	0.074	-	-	-
**Body weight (kg)**	−0.273	−0.672 to 0.125	0.182	-	-	-
**Serum creatinine (µmol/L)**	0.035	−0.062 to 0.132	0.475	-	-	-
**The forecast lead time (day)**	−3.333	−3.928 to −2.738	<0.001	−3.311	−3.902 to −2.720	<0.001
**Vancomycin concentration**						
Two concentrations	Reference					
Mid concentration	1.343	−2.544 to 5.229	0.498	0.618	−3.235 to 4.470	0.753
Peak concentration	−1.961	−3.510 to −0.411	0.013	−1.298	−2.868 to 0.272	0.105
Trough concentration	−2.537	−4.101 to −0.974	0.001	−1.847	−3.429 to −0.264	0.022
**Estimation methods**						
Conventional Bayesian method	Reference					
Flattened coefficient 0.005	3.472	0.921 to 6.023	0.008	3.472	0.941 to 6.003	0.007
Flattened coefficient 0.02	3.395	0.845 to 5.946	0.009	3.395	0.864 to 5.927	0.009
Flattened coefficient 0.125	2.945	0.394 to 5.496	0.024	2.945	0.414 to 5.476	0.023
Flattened coefficient 0.2	2.546	−0.004 to 5.097	0.051	2.546	0.015 to 5.078	0.049
Flattened coefficient 0.3	2.078	−0.473 to 4.629	0.110	2.078	−0.453 to 4.610	0.108
Flattened coefficient 0.6	1.022	−1.529 to 3.572	0.433	1.022	−1.510 to 3.553	0.429
Weighted-flattened Bayesian method	12.662	10.111 to 15.213	<0.001	12.660	10.131 to 15.194	<0.001
First-order PK	9.900	6.293 to 13.508	<0.001	8.932	5.242 to 12.623	<0.001

**Table 3:** Results of the linear mixed-effect regression analysis for the dependent variable rRMSE.

Variables	Unadjusted			Adjusted		
	Estimate	95% CI	*p*	Estimate	95% CI	*p*
**Age (month)**	0.087	0.023 to 0.151	0.009	0.123	0.049 to 0.199	0.002
**Body weight (kg)**	0.187	−0.052 to 0.425	0.128	-	-	-
**Serum creatinine (µmol/L)**	−0.210	−0.282 to −0.139	<0.001	−0.252	−0.325 to −0.181	<0.001
**The forecast lead time (day)**	3.187	2.792 to 3.582	<0.001	3.227	2.834 to 3.621	<0.001
**Vancomycin concentration**						
Two concentrations	Reference					
Mid concentration	1.804	−0.789 to 4.397	0.173	3.523	0.960 to 6.086	0.007
Peak concentration	5.501	4.467 to 6.536	<0.001	5.402	4.360 to 6.443	<0.001
Trough concentration	−0.012	−1.054 to 1.033	0.984	−0.204	−1.255 to 0.847	0.703
**Estimation methods**						
Conventional Bayesian method	Reference					
Flattened coefficient 0.005	0.537	−1.191 to 2.265	0.543	0.537	−1.143 to 2.218	0.531
Flattened coefficient 0.02	0.378	−1.350 to 2.106	0.668	0.378	−1.302 to 2.059	0.659
Flattened coefficient 0.125	0.049	−1.679 to 1.777	0.956	0.049	−1.632 to 1.729	0.954
Flattened coefficient 0.2	−0.103	−1.830 to 1.625	0.907	−0.103	−1.783 to 1.578	0.905
Flattened coefficient 0.3	−0.254	−1.981 to 1.474	0.774	−0.254	−1.934 to 1.427	0.767
Flattened coefficient 0.6	−0.370	−2.098 to 1.358	0.675	−0.370	−2.051 to 1.310	0.666
Weighted-flattened Bayesian method	−2.099	−3.826 to −0.371	0.017	−2.099	−3.779 to −0.418	0.014
First-order PK	−2.440	−4.883 to 0.003	0.050	−0.653	−3.103 to 1.797	0.601

## Data Availability

The data files supporting the reported results were found in the Supplementary Materials. Code availability, including the model control streams in the data analysis, was also included.

## Supplementary Materials

The following supporting information can be downloaded at: https://www.mdpi.com/article/10.3390/pharmaceutics18020160/s1, Table S1: Data collection form; Model control streams applied for Bayesian algorithms; Data files supporting the reported results, including forecast_2pts.csv, forecast_peak.csv, forecast_mid.csv, forecast_trough.csv, and lmer_test_data.csv.

## Abbreviations

The following abbreviations are used in this manuscript:

PK	Pharmacokinetics
TDM	Therapeutic Drug Monitoring
rBias	Relative Bias
RMSE	Relative Root Mean Squared Error
MRSA	Methicillin-Resistant *Staphylococcus aureus*
AKI	Acute Kidney Injury
popPK	Population Pharmacokinetics
AUC	Area Under the Curve
PEX	Plasma Exchange
ECMO	Extracorporeal Membrane Oxygenation
eGFR	Estimated Glomerular Filtration Rate
ICU	Intensive Care Unit
MAP	Maximum A Posteriori
IIV	Individual Variabilities
MPID	Model-Informed Precision Dosing
