# Supplementary material for: Predictive Performance of Bayesian Methods to Forecast Vancomycin Concentration for Therapeutic Drug Monitoring in Critically Ill Pediatric Patients
Source: Pharmaceutics. 2026 Jan 26;18(2):160. doi: 10.3390/pharmaceutics18020160 (PMC12944451; doi:10.3390/pharmaceutics18020160)
Supplement: Supplementary file 1 [file pharmaceutics-18-00160-s001.zip › pharmaceutics-4081448-supplementary-R1.pdf]

pharmaceutics

MDPI

Supplementary Materials

PREDICTIVE PERFORMANCE OF BAYESIAN METHODS TO FORECAST VANCOMYCIN CONCENTRATION FOR THERAPEUTIC DRUG MONITORING IN CRITICALLY ILL PEDIATRIC PATIENTS

Ha T. Pham, Cuc T. Nguyen, Tien T. N. Nguyen, Linh H. Hoang, Minh N. Tran, Thao P. Nguyen, Tuan N. Do, Ha T. H. Nguyen, Anh H. Nguyen, Phuc H. Phan, Dien M. Tran and Hoa D. Vu

The supplementary materials include the following contents:

1, Table S1. Data collection form

2, Model control streams applied for Bayesian algorithms

3, Data files supporting the reported results

The data files supporting the reported results include: **forecast_2pts.csv**, **forecast_peak.csv**, **forecast_mid.csv**, **forecast_trough.csv**, and **lmer_test_data.csv**.

- The "**forecast_2pts.csv**", "**forecast_peak.csv**", "**forecast_mid.csv**", and "**forecast_trough.csv**" files are the datasets input into the forecasting analysis, including the data of patient ID, time (vancomycin administration time, sampling time), infusion rate, observed vancomycin concentrations, and patient characteristics (age, weight, serum creatinine).
- The "**forecast_2pts.csv**" file includes two concentrations in the preceding TDM of each patient to estimate individual PK parameters and forecast concentrations in the next TDM. Meanwhile, the "**forecast_peak.csv**", "**forecast_mid.csv**", or "**forecast_trough.csv**" includes either only peak, mid, or trough concentration in the preceding TDM.
- The "**lmer_test_data.csv**" file is the dataset input into the linear mixed effect regression to identify factors associated with the forecast performance, which was indirectly represented by rBias and rRMSE. The "**lmer_test_data.csv**" file includes the data of patient ID, Cforecast by the different methods, Cobserved, the respective rBias and rRMSE values, the type of concentrations used, the type of algorithms applied, the forecast lead time, and patient characteristics (age, weight, and serum creatinine).

Table S1. Data collection form

VANCOMYCIN THERAPEUTIC DRUG MONITORING

Internal Medicine Intensive Care Unit, Vietnam National Children's Hospital

**I. Patient's characteristics**
Full name:.. Medical Record Number (PRN):..
Date of birth:............................ Height:...................... Body weight: .... Gender: ☐Male ☐Female
Outcomes: ☐Recovered ☐Improved ☐Unchanged ☐Worsening ☐Deceased
Date of hospitalization .... Date of hospital discharge..   Date of ICU admission .... Date of ICU discharge...
Major/Infection diagnosis: ....   Comorbidities... ☐ Preterm
Interventions/Surgeries: • Renal Replacement Therapy (RRT):   ☐ No ☐CRRT ☐ IHD ☐ Peritoneal dialysis   Duration of RRT (if any): ....   • Mechanical ventilation (MV): ☐ No ☐Yes Duration of MV (if any): ....   • ECMO: ☐ No ☐Yes Treatment duration : ....

PIM 3 Score:	
1. Systolic blood pressure (mmHg) before ICU admission (No information → 120):...	5. Using mechanical ventilation during the first hours in ICU? ☐ No ☐Yes
2. Pupillary reflex when hospitalised: Pupil >3mm AND fixed Others:... No information:	6. Was ICU admission scheduled? ☐No ☐ Yes 7. Was postoperative management the main cause for ICU admission? ☐ No ☐ Yes, after cardiovascular interventions WITH bypass grafting ☐ Yes, after cardiovascular interventions WITHOUT bypass grafting ☐ Yes, after non-cardiovascular interventions
3. FiO2 (%): .... PaO2 (mmHg):... (Note: Collecting these two indices simultaneously within the first hour after ICU admission)	
4. Base excess (BE) (mmol/L):	

II. Co-medication

Concomitant drugs	Treatment duration	Concomitant drugs	Treatment duration

III. Clinical laboratory results

Microbiological test

Specimen	Sampling date	Results release date	Results (negative/positive, pathogen isolated)	MIC vancomycin

Other

**Date**						
**Albumin (g/L)**						
**Date**						
**BUN (μmol/L)**						
**Date**						
**UO (mg/kg/h)**						
**Date**						
**Bilan 24h (mL)**						

IV. Vancomycin administration and therapeutic drug monitoring characteristics

Vancomycin duration of therapy (start date – end date):………

Solution for infusion: ……………………………………………… **Infusion volume:**………

Date	Dose (mg)	Infusion duration (min)	Infusion start time (…h…min)				Scr (µmol/L)	Cpeak sampling time	Cpeak (mg/L)	Ctrough sampling time	Ctrough (mg/L)
			1st dose	2nd dose	3rd dose	4th dose					

Model control streams applied for Bayesian algorithms

Model control stream when using the conventional Bayesian approach (i.e., the standard Maximum A Posteriori estimation) implemented in NONMEM software presented below.

```
$PROBLEM Le model - literature model
;; 2. Description:
;; x1. Author: DIADR
;; 3. Label:
;----------------------------------
$INPUT ID TIME AMT EVID RATE DV typeC=DROP WT AGE SCR TDM_order conc
;----------------------------------
$DATA    forecast_2pts.csv IGNORE=@
;----------------------------------
$SUBROUTINES ADVAN1 TRANS2
;----------------------------------
$PK
;----------------------------------
TVCL = THETA(1) * (WT**0.75) *((0.4/SCR)**0.431)*((log(AGE)/7.7)**0.808)
CL = TVCL * EXP(ETA(1))
TVV = THETA(2) * WT
V = TVV * EXP(ETA(2))
S1 = V
;----------------------------------
$THETA
0.258 FIX    ; CL
0.644 FIX    ; V
;----------------------------------
$OMEGA BLOCK (2) FIX
0.1681              ; eta1
0.001968              0.0144; eta2
;----------------------------------
$SIGMA
0.1024 FIX ;PROP
$ERROR
;----------------------------------
IPRED = F
IRES = DV-IPRED
W = F
IWRES = IRES/W
Y= IPRED+W*EPS(1)
;----------------------------------
$EST METHOD= 1 INTERACTION MAXEVAL=0 NOABORT SIG=3 PRINT=5
;----------------------------------
$COV PRINT=E UNCONDITIONAL
```

```
;----------------------------------
;----------------------------------
; Xpose
$TABLE ID EVID TIME TDM_order IPRED conc NOPRINT ONEHEADER NOPRINT FILE = output_forecast_2pts
$TABLE ID CL V NOPRINT ONEHEADER FILE = Indiv_param_2pts
```

Model control streams when using the flattened Bayesian approach with the flat_coef of 0.005, 0.02, 0.125, 0.2, 0.3, and 0.6 implemented in NONMEM software presented below.

```
$PROBLEM Le model - literature model
;; 2. Description:
;; x1. Author: DIADR
;; 3. Label:
;----------------------------------
$INPUT ID TIME AMT EVID RATE DV typeC=DROP WT AGE SCR TDM_order conc
;----------------------------------
$DATA    forecast_2pts.csv IGNORE=@
;----------------------------------
$SUBROUTINES ADVAN1 TRANS2
;----------------------------------
$PK
;----------------------------------
TVCL = THETA(1) * (WT**0.75) *((0.4/SCR)**0.431)*((log(AGE)/7.7)**0.808)
CL = TVCL * EXP(ETA(1))
TVV = THETA(2) * WT
V = TVV * EXP(ETA(2))
S1 = V
;----------------------------------
$THETA
0.258 FIX    ; CL
0.644 FIX    ; V
;----------------------------------
$OMEGA BLOCK (2) FIX
33.62              ; eta1
0.3936              2.88; eta2
;----------------------------------
$SIGMA
0.1024 FIX ;PROP
$ERROR
;----------------------------------
IPRED = F
IRES = DV-IPRED
W = F
IWRES = IRES/W
```

```
Y= IPRED+W*EPS(1)
;----------------------------------
$EST METHOD= 1 INTERACTION MAXEVAL=0 NOABORT SIG=3 PRINT=5
;----------------------------------
$COV PRINT=E UNCONDITIONAL
;----------------------------------
;----------------------------------
; Xpose
$TABLE ID EVID TIME TDM_order IPRED conc NOPRINT ONEHEADER NOPRINT FILE = output_forecast_2pts
$TABLE ID CL V NOPRINT ONEHEADER FILE = Indiv_param_2pts

$PROBLEM Le model - literature model
;; 2. Description:
;; x1. Author: DIADR
;; 3. Label:
;----------------------------------
$INPUT ID TIME AMT EVID RATE DV typeC=DROP WT AGE SCR TDM_order conc
;----------------------------------
$DATA    forecast_2pts.csv IGNORE=@
;----------------------------------
$SUBROUTINES ADVAN1 TRANS2
;----------------------------------
$PK
;----------------------------------
TVCL = THETA(1) * (WT**0.75) *((0.4/SCR)**0.431)*((log(AGE)/7.7)**0.808)
CL = TVCL * EXP(ETA(1))
TVV = THETA(2) * WT
V = TVV * EXP(ETA(2))
S1 = V
;----------------------------------
$THETA
0.258 FIX    ; CL
0.644 FIX    ; V
;----------------------------------
$OMEGA BLOCK (2) FIX
8.405              ; eta1
0.0984             0.72; eta2
;----------------------------------
$SIGMA
0.1024 FIX ;PROP
$ERROR
;----------------------------------
IPRED = F
IRES = DV-IPRED
```

```
W = F
IWRES = IRES/W
Y= IPRED+W*EPS(1)
;----------------------------------
$EST METHOD= 1 INTERACTION MAXEVAL=0 NOABORT SIG=3 PRINT=5
;----------------------------------
$COV PRINT=E UNCONDITIONAL
;----------------------------------
;----------------------------------
; Xpose
$TABLE ID EVID TIME TDM_order IPRED conc NOPRINT ONEHEADER NOPRINT FILE = output_forecast_2pts
$TABLE ID CL V NOPRINT ONEHEADER FILE = Indiv_param_2pts

$PROBLEM Le model - literature model
;; 2. Description:
;; x1. Author: DIADR
;; 3. Label:
;----------------------------------
$INPUT ID TIME AMT EVID RATE DV typeC=DROP WT AGE SCR TDM_order conc
;----------------------------------
$DATA    forecast_2pts.csv IGNORE=@
;----------------------------------
$SUBROUTINES ADVAN1 TRANS2
;----------------------------------
$PK
;----------------------------------
TVCL = THETA(1) * (WT**0.75) *((0.4/SCR)**0.431)*((log(AGE)/7.7)**0.808)
CL = TVCL * EXP(ETA(1))
TVV = THETA(2) * WT
V = TVV * EXP(ETA(2))
S1 = V
;----------------------------------
$THETA
0.258 FIX    ; CL
0.644 FIX    ; V
;----------------------------------
$OMEGA BLOCK (2) FIX
1.3448              ; eta1
0.015744             0.1152; eta2
;----------------------------------
$SIGMA
0.1024 FIX ;PROP
$ERROR
;----------------------------------
```

```
IPRED = F
IRES = DV-IPRED
W = F
IWRES = IRES/W
Y= IPRED+W*EPS(1)
;----------------------------------
$EST METHOD= 1 INTERACTION MAXEVAL=0 NOABORT SIG=3 PRINT=5
;----------------------------------
$COV PRINT=E UNCONDITIONAL
;----------------------------------
;----------------------------------
; Xpose
$TABLE ID EVID TIME TDM_order IPRED conc NOPRINT ONEHEADER NOPRINT FILE = output_forecast_2pts
$TABLE ID CL V NOPRINT ONEHEADER FILE = Indiv_param_2pts

$PROBLEM Le model - literature model
;; 2. Description:
;; x1. Author: DIADR
;; 3. Label:
;----------------------------------
$INPUT ID TIME AMT EVID RATE DV typeC=DROP WT AGE SCR TDM_order conc
;----------------------------------
$DATA    forecast_2pts.csv IGNORE=@
;----------------------------------
$SUBROUTINES ADVAN1 TRANS2
;----------------------------------
$PK
;----------------------------------
TVCL = THETA(1) * (WT**0.75) *((0.4/SCR)**0.431)*((log(AGE)/7.7)**0.808)
CL = TVCL * EXP(ETA(1))
TVV = THETA(2) * WT
V = TVV * EXP(ETA(2))
S1 = V
;----------------------------------
$THETA
0.258 FIX    ; CL
0.644 FIX    ; V
;----------------------------------
$OMEGA BLOCK (2) FIX
0.8405              ; eta1
0.00984              0.072; eta2
;----------------------------------
$SIGMA
0.1024 FIX ;PROP
```

```
$ERROR
;----------------------------------
IPRED = F
IRES = DV-IPRED
W = F
IWRES = IRES/W
Y= IPRED+W*EPS(1)
;----------------------------------
$EST METHOD= 1 INTERACTION MAXEVAL=0 NOABORT SIG=3 PRINT=5
;----------------------------------
$COV PRINT=E UNCONDITIONAL
;----------------------------------
;----------------------------------
; Xpose
$TABLE ID EVID TIME TDM_order IPRED conc NOPRINT ONEHEADER NOPRINT FILE = output_forecast_2pts
$TABLE ID CL V NOPRINT ONEHEADER FILE = Indiv_param_2pts

$PROBLEM Le model - literature model
;; 2. Description:
;; x1. Author: DIADR
;; 3. Label:
;----------------------------------
$INPUT ID TIME AMT EVID RATE DV typeC=DROP WT AGE SCR TDM_order conc
;----------------------------------
$DATA    forecast_2pts.csv IGNORE=@
;----------------------------------
$SUBROUTINES ADVAN1 TRANS2
;----------------------------------
$PK
;----------------------------------
TVCL = THETA(1) * (WT**0.75) *((0.4/SCR)**0.431)*((log(AGE)/7.7)**0.808)
CL = TVCL * EXP(ETA(1))
TVV = THETA(2) * WT
V = TVV * EXP(ETA(2))
S1 = V
;----------------------------------
$THETA
0.258 FIX    ; CL
0.644 FIX    ; V
;----------------------------------
$OMEGA BLOCK (2) FIX
0.56033              ; eta1
0.006559              0.048; eta2
;----------------------------------
```

```
$SIGMA
0.1024 FIX ;PROP
$ERROR
;----------------------------------
IPRED = F
IRES = DV-IPRED
W = F
IWRES = IRES/W
Y= IPRED+W*EPS(1)
;----------------------------------
$EST METHOD= 1 INTERACTION MAXEVAL=0 NOABORT SIG=3 PRINT=5
;----------------------------------
$COV PRINT=E UNCONDITIONAL
;----------------------------------
;----------------------------------
; Xpose
$TABLE ID EVID TIME TDM_order IPRED conc NOPRINT ONEHEADER NOPRINT FILE = output_forecast_2pts
$TABLE ID CL V NOPRINT ONEHEADER FILE = Indiv_param_2pts

$PROBLEM Le model - literature model
;; 2. Description:
;; x1. Author: DIADR
;; 3. Label:
;----------------------------------
$INPUT ID TIME AMT EVID RATE DV typeC=DROP WT AGE SCR TDM_order conc
;----------------------------------
$DATA    forecast_2pts.csv IGNORE=@
;----------------------------------
$SUBROUTINES ADVAN1 TRANS2
;----------------------------------
$PK
;----------------------------------
TVCL = THETA(1) * (WT**0.75) *((0.4/SCR)**0.431)*((log(AGE)/7.7)**0.808)
CL = TVCL * EXP(ETA(1))
TVV = THETA(2) * WT
V = TVV * EXP(ETA(2))
S1 = V
;----------------------------------
$THETA
0.258 FIX    ; CL
0.644 FIX    ; V
;----------------------------------
$OMEGA BLOCK (2) FIX
0.280167              ; eta1
```

```
0.00328            0.024; eta2
;----------------------------------
$SIGMA
0.1024 FIX ;PROP
$ERROR
;----------------------------------
IPRED = F
IRES = DV-IPRED
W = F
IWRES = IRES/W
Y= IPRED+W*EPS(1)
;----------------------------------
$EST METHOD= 1 INTERACTION MAXEVAL=0 NOABORT SIG=3 PRINT=5
;----------------------------------
$COV PRINT=E UNCONDITIONAL
;----------------------------------
;----------------------------------
; Xpose
$TABLE ID EVID TIME TDM_order IPRED conc NOPRINT ONEHEADER NOPRINT FILE = output_forecast_2pts
$TABLE ID CL V NOPRINT ONEHEADER FILE = Indiv_param_2pts
```

In the $DATA section, "**forecast_2pts.csv**" was the input dataset, including two concentrations in the preceding TDM, in order to estimate individual PK parameters for each patient and forecast concentrations in the next TDM. The text "**forecast_2pts.csv**" was replaced by "**forecast_peak.csv**", "**forecast_mid.csv**", or "**forecast_trough.csv**" when only one of these concentrations was used.

In the $TABLE sections, "**Indiv_forecast_2pts**" and "**output_forecast_2pts**" represent output files related to the individual PK parameters estimated and the concentration in the next TDM forecasted from two previous concentrations, respectively. The text "**Indiv_forecast_2pts**" was replaced by "**Indiv_forecast_peak**", "**Indiv_forecast_mid**", or "**Indiv_forecast_trough**", and the text "**output_forecast_2pts**" was also replaced by "**output_forecast_peak**", "**output_forecast_mid**", or "**output_forecast_trough**", depending on the respective input datasets used.
